# Supplementary material for: Transcriptomic Sequencing Analysis on Key Genes and Pathways Regulating Cadmium (Cd) in Ryegrass (Lolium perenne L.) under Different Cadmium Concentrations
Source: Toxics. 2022 Nov 28;10(12):734. doi: 10.3390/toxics10120734 (PMC9782025; doi:10.3390/toxics10120734)
Supplement: Supplementary file 1 [file toxics-10-00734-s001.zip › toxics-1965342-supplementary materials.pdf]

Table S1. The growth and physiological indicators of ryegrass.

Treatment	Height (cm)	Weight (g)	Tiller numbers	Soluble protein (μg·g^{-1} FW)	Chla (mg·g^{-1}FW)
C0	65.07±2.20a	17.19±1.60a	6.33±0.52a	242.67±16.36b	2.68±0.48a
C5	60.17±1.57ab	13.79±1.13b	5.00±0.89a	252.78±19.47ab	3.03±0.38a
C25	56.57±1.66ab	13.47±1.41b	5.67±1.37a	282.78±12.71a	3.10±0.23a
C50	53.57±4.29bc	12.17±0.82b	5.33±0.52a	252.28±13.43ab	2.96±0.32a
C100	53.67±2.44cd	12.44±1.03b	4.67±0.52ab	232.83±20.63b	3.03±0.52a
C500	48.77±3.19d	5.00±0.41c	3.00±0.89b	261.44±11.79ab	3.15±0.39a
F	10.531	30.200	4.350	2.716	0.427
P	< 0.001	< 0.001	0.017	0.073	0.821

Note: Data are expressed as mean values with standard deviations (±SD), and analyzed by ANOVA with Duncan's test. Different lowercase letters indicate that values are significantly different at $P < 0.05$.

Table S2. Enzyme activity varying in ryegrass.

Treatment	MDA (nmol·g^{-1} FW)	SOD (U·g^{-1} FW)	POD (U·g^{-1} FW)	CAT (U·g^{-1} FW)
C0	27.83±2.31b	37.41±3.55d	76.17±13.34c	4.50±0.82c
C5	19.37±3.14d	208.78±69.10c	109.17±11.90b	5.20±1.42c
C25	19.90±2.12cd	254.69±46.08c	148.67±8.85a	7.80±0.54a
C50	17.92±2.41d	513.59±110.79b	135.00±29.33ab	6.23±0.05abc
C100	24.77±0.14bc	812.13±131.42a	108.00±12.23b	7.37±0.69ab
C500	33.78±3.50a	338.16±47.12c	133.92±8.85ab	5.97±0.69bc
F	14.202	27.291	6.608	5.731
P	< 0.001	< 0.001	0.004	0.006

Note: Data are expressed as mean values with standard deviations (±SD), and analyzed by ANOVA with Duncan's test. Different lowercase letters indicate that values are significantly different at $P < 0.05$.

Table S3. Antioxidant enzyme gene expression abundance in ryegrass under cadmium stress.

Treatment	*POD*	*CAT*	*APX*	*Cu/ZnSOD*	*FeSOD*	*MnSOD*
C0	1.03±0.28b	1.00±0.00c	1.75±0.12b	1.11±0.54b	1.03±0.31c	1.04±0.34ab
C5	4.35±2.00b	2.77±1.98c	0.22±0.14c	0.57±0.33c	1.21±0.58bc	1.30±0.64a
C25	25.34±13.60a	1.37±0.87c	1.47±0.02b	0.86±0.18bc	1.12±0.62bc	1.45±0.26a
C50	2.05±1.28b	1.62±0.23c	2.88±0.41a	2.30±0.63a	2.58±0.50b	1.30±0.16a
C100	0.13±0.03b	10.36±3.87b	1.45±0.12b	0.54±0.16bc	2.52±0.53b	0.78±0.08b
C500	0.85±0.16b	15.54±5.13a	3.34±0.61a	2.47±0.57a	4.17±1.47a	0.97±0.17ab
F	9.013	14.429	37.741	13.452	7.826	2.719
P	0.001	< 0.001	<0.001	< 0.001	0.002	0.072

Note: Data are expressed as mean values with standard deviations (±SD), and analyzed by ANOVA with Duncan's test. Different lowercase letters indicate that values are significantly different at $P < 0.05$.

Table S4. Summary of sequencing data statistics.

Sample	Clean reads	Clean bases	Error rate (%)	Q20 (%)	Q30 (%)	GC content (%)
C0_1	46,641,088	6,842,055,349	0.0247	98.11	94.50	55.16
C0_2	47,641,532	6,974,942,419	0.0245	98.21	94.72	55.32
C0_3	43,093,830	6,339,134,777	0.0247	98.11	94.47	54.97
C50_1	47,116,950	6,910,700,655	0.0245	98.19	94.68	56.23
C50_2	50,036,728	7,364,445,126	0.0244	98.22	94.74	55.05
C50_3	43,855,802	6,519,257,208	0.0244	98.24	94.79	55.54
C500_1	51,212,352	7,403,707,950	0.0245	98.21	94.71	55.68
C500_2	50878,358	7381652,488	0.0245	98.20	94.68	54.31
C500_3	46455,420	6794815,811	0.0246	98.15	94.60	55.20

Table S5. Optimized transcriptome assembly details.

Type	Unigene	Transcript
Total number	118,443	222,269
Total sequence base	89,265,563	183,347,587
Largest length (bp)	15,667	15,667
Smallest length (bp)	201	201
Average length (bp)	754	825
N50 length (bp)	1168	1223
E90N50 length (bp)	2091	1725
Mean mapped percent (%)	73.905	83.512
GC percent (%)	49.34	50.06
TransRate score	0.1691	0.22442
BUSCO score	78.5% (1.1%)	78.5% (1.1%)

Table S6. Alignment and quality analysis of transcriptome data.

Sample	Clean reads	Mapped reads	Mapped ratio
C0_1	46,641,088	33,855,478	72.59%
C0_2	47,641,532	34,550,862	72.52%
C0_3	43,093,830	30,729,218	71.31%
C50_1	47,116,950	33,972,838	72.10%
C50_2	50,036,728	36,057,502	72.06%
C50_3	43,855,802	31,191,086	71.12%
C500_1	51,212,352	37,085,298	72.41%
C500_2	50,878,358	37,188,322	73.09%
C500_3	46,455,420	33,450,956	72.01%

Table S7. Unigenes annotation results from six databases.

	Unigene number	Percent (%)
NR	52,373	44.22
Swiss-Prot	32,703	27.61
Pfam	32,907	27.78
COG	48,414	40.88
GO	43,497	36.72
KEGG	20,654	17.44
Total_anno	53,550	45.21
Total	118,443	100

Table S8. Primers used for qRT-PCR.

Gene ID	Primer sequences (5'to3')	Product size (bp)
DN12652_c0_g1	F: AGGAAATGATGGACAGCGGG<br>R: ACAGTTGGGCACTACCTCCT	103
DN5585_c0_g1	F: CGAGGAGGACAGATAGCTCG<br>R: TGAAATCCACGGAAGCCACT	90
DN123028_c0_g1	F: GCAAAGATGGATGCCTCGGA<br>R: TGCAATGGCTGTCCTTGACT	99
DN92214_c0_g2	F: TGACCGTATGCCATCTGCAC<br>R: TTTACAGACGACGGCGACAA	93
DN18566_c0_g1	F: GATCGAGGCCTGGGATGAAC<br>R: TTCAGAAGATCAGCCGACCG	92
DN85_c0_g1	F: ATATGCCCGTCTACCTCCCA<br>R: TTGGGTTTCGCTACGGAGTC	92
DN18177_c0_g1	F: CACGAACACGACATGACATT<br>R: AAATTACGGGAGCATGCGTT	120
DN290_c0_g3	F: ACAAACACCAACCTCGGCA<br>R: TGTGGGATGTCGATCACCAG	107
DN4816_c0_g1	F: CAAAGCGATCGGATTCGCAG<br>R: GCTGGTCCTCTGCATCCATT	92
DN75_c0_g1	F: TCGCGTTCTCTATCCGCTTC<br>R: TTGCCACTTCCGTTCCGAAT	95

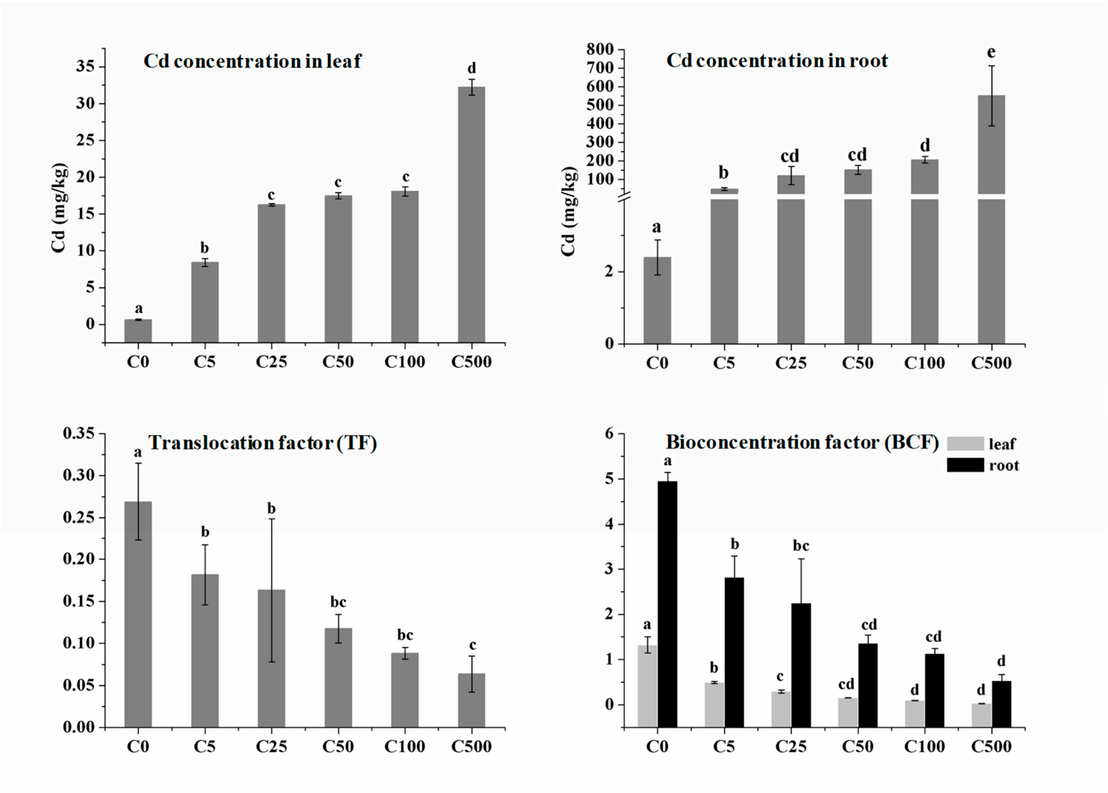

Figure S1. Accumulation of Cd content in different parts of ryegrass.

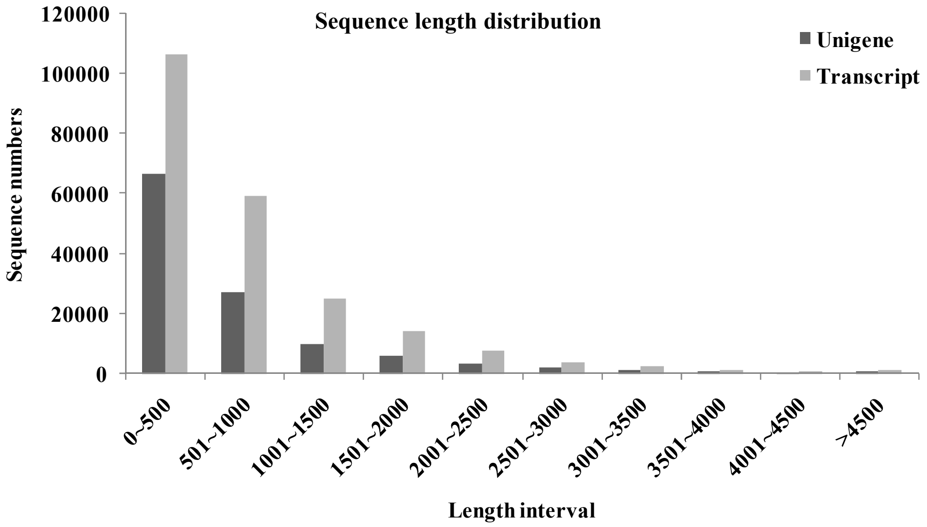

Figure S2. The distribution of unigenes and transcripts retrieving from ryegrass transcriptome.

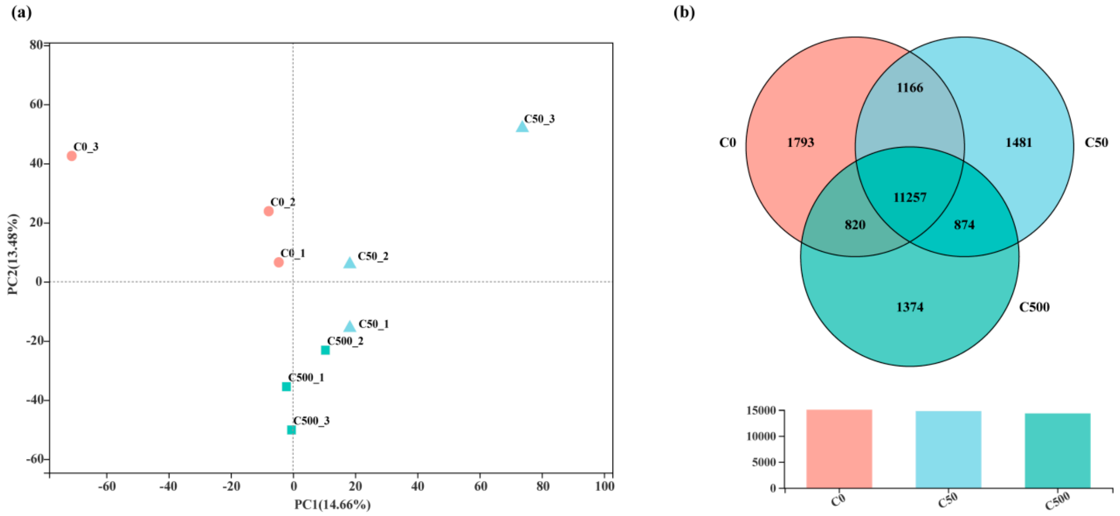

Figure S3. (a) Principal component analysis (PCA) of expression genes in different treatments. (b) Venn diagram of expression genes among three treatments.

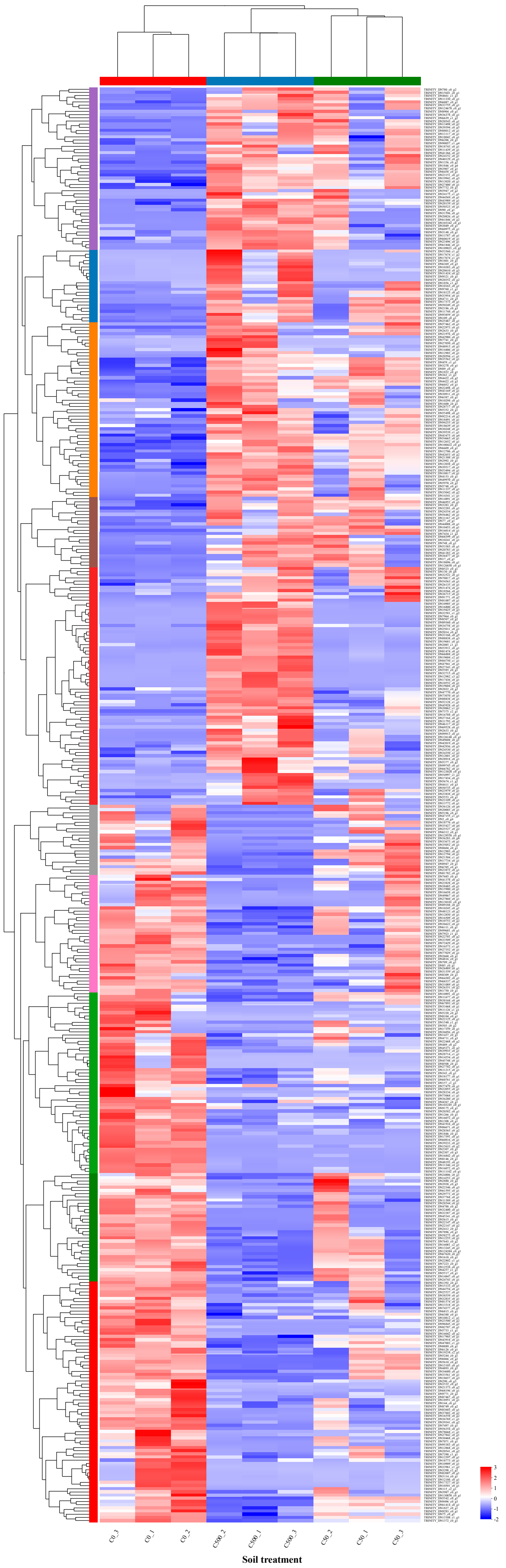
C0_3
C0_1
C0_2
C500_2
C500_1
C500_3
C50_2
C50_1
C50_3
Soil treatment
3
2
1
0
-1
-2

Figure S4. Cluster analysis of differentially expressed genes. Note: Each line represented an unigene. Red represented the high expression level of the unigene in the sample, blue represented the low expression level. On the left is a dendrogram of unigene clustering. Branches with similar expression of unigene are clustered together.

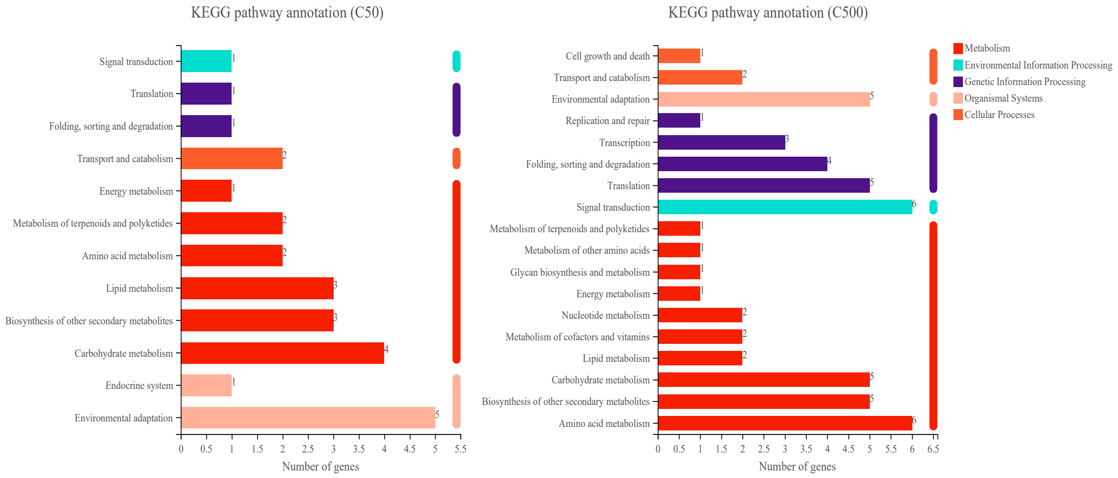

Figure S5. KEGG analysis of DEGs in ryegrass leaves among different treatments.

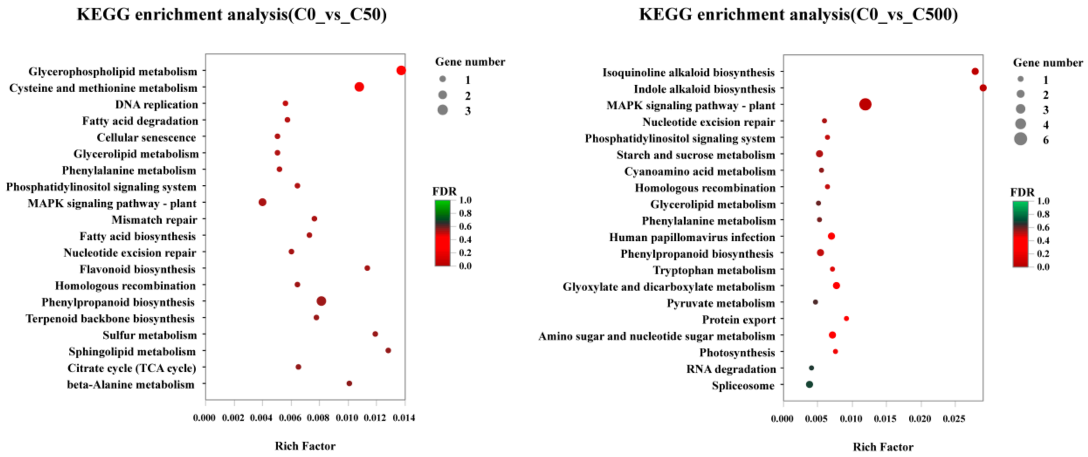

Figure S6. KEGG enrichment analysis of significantly regulated genes in ryegrass leaves under Cd stress.
